# Forage agronomists are needed in animal science departments

**DOI:** 10.1093/tas/txac019

**Published:** 2022-01-29

**Authors:** Michael L Looper, John A Jennings, J Daniel Rivera

**Affiliations:** 1 Department of Animal Science, University of Arkansas System Division of Agriculture, Fayetteville, AR 72701, USA; 2 Department of Animal Science, University of Arkansas System Division of Agriculture, Little Rock, AR 72204, USA; 3 Department of Animal Science, University of Arkansas System Division of Agriculture, Southwest Research and Extension Center, Hope, AR 71801, USA

**Keywords:** animal science, forage agronomy, graduate program, teaching

## Abstract

Ruminants serve a valuable role in sustainable agricultural systems, specifically in the conversion of renewable resources from grasslands, pasture, and other by-products into high-quality human food. Recognizing forage and grasses are grown on 25% of arable land, suitable agronomic practices for management of grazing livestock are necessary for the economic sustainability of the livestock enterprise, whereas at the same time, minimizing water and soil erosion. Demographics of undergraduate animal science students have changed over the last several years with more students from urban backgrounds and with interests other than traditional animal agriculture. Thus, continued emphasis on education programs supporting grazing livestock industries becomes that much more important. In addition, newer technologies to measure production on range and pastureland have emerged, thereby increasing opportunities for further training and education. Based on an email assessment of 10 land grant institutions, typically one MS student/yr and one PhD student/3 to 4 yr graduates with an advanced degree in forage agronomy. Overall budget reductions which impact operational costs, internal funding for research projects and graduate student stipends, force universities to focus in areas with the best chance of monetary return. Challenges with funding faculty positions outside of a department’s emphasis area typically result in the question “Should forage agronomy students be trained in Departments of Animal Science or Agronomy/Plant/Soils Sciences?” It could be argued that either department is the best fit. Forage agronomy requires training in the basics of plant and soil science, but the application of those sciences within a Department of Animal Science relates more to animal science/production than to traditional crop production such as cereal grains. Animal science departments must communicate the meaningful context of forage agronomy in an active learning environment developing students’ ability to critically think and solve problems. Those providing technical expertise to livestock producers can no longer make recommendations based solely on production efficiency and profitability. Instead, best management practices must include the impact of grazing livestock on the environment and environmental sustainability. Cooperative agreements between departments should be discussed to adequately support student development in this critical subject matter.

## INTRODUCTION

Agricultural land area is approximately five billion hectares or 38% of the global land surface (FAO, 2020). About 1/3 is used as cropland with the remaining 2/3 of land area consisting of meadows and pastures for grazing livestock (Oltjen and Beckett, 1996). Global cropland area per capita decreased between 1961 and 2016 from 0.45 ha/capita in 1961 to 0.21 ha/capita in 2016 (FAO, 2020). Although expected estimates of global population growth are inconsistent, there seems to be a clear need to improve productive of cropland to meet nutritional needs of the masses, specifically in developing countries such as sub-Saharan Africa, India, and Indonesia (Adam, 2021). To accomplish the arduous task of feeding the world, training of experts that comprehend the livestock-grazing land interface is necessary.

Ruminants convert products of grazing lands not readily suited for agronomic crop production, as well as crop residues/by-products into value added products (Oltjen and Beckett, 1996); only 2.8 kg of human-edible feed is necessary to produce 1 kg of boneless meat (Mottet et al., 2017). Ruminants are responsible for most all of the grassland forage conversion to milk and 29% of grassland forage conversion to meat worldwide (Gerber et al., 2013). Livestock production (e.g., enteric fermentation) accounts for approximately 2.5% of total U.S. greenhouse gas (GHG) emissions (EPA, 2019). Simulation models removing all animal-derived foods from the U.S. agricultural system resulted in decreased GHG emissions of 2.65% (White and Hall, 2017). However, these modeled human diets were nonviable to support nutrient needs without additional supplementation.

Challenges exist for researchers to conduct statistically relevant studies in grazing systems due to lack of resources (cattle and land availability) as well as variable environmental issues (rainfall, snowfall, droughts, and pests). In addition to the limited resources, finding suitable replicated units as well as designing studies that will mimic the production environment can limit success of researchers to publish data in meaningful peer reviewed journals (DelCurto and Wyffels, 2021). These concerns often make it difficult to attract graduate students to such programs. Roles of a forage agronomist are vast and require expertise in the plant-animal interface developing knowledge and guidelines in, but not limited to chemical, physical, and botanical characteristics of forage plants, animal nutrition, plant physiology, forage establishment, fertilization, pest management, harvest and storage, grazing management, soil-plant interactions, alternative forage crops, and integration of livestock with crop systems.

A continued emphasis on education programs supporting the grazing livestock industry is more important now than ever. Increased emphasis on sustainable agricultural practices, more niche type markets (e.g., pasture-raised and grass-fed beef), and tighter production margins requires trained individuals that understand the forage/livestock nutritional interaction. Nonetheless, for some of the previously stated reasons, land grant universities (LGU) have placed less emphasis on training PhD students in forage agronomy (Rouquette et al., 2009). With fewer university positions within forage agronomy, there is less emphasis on student training within this field further exacerbating this conundrum. Our commentary focuses on the current state of training forage agronomists in the Southeastern United States and thoughts on why so few are being trained. Furthermore, we provide justification of forage agronomists’ placement and faculty home in the animal sciences.

## MATERIALS AND METHODS

### Assessment of Land Grant Universities

An email assessment was conducted of the list serve of LGU participating in the Southern Pasture & Forage Crop Improvement Conference (SPFCIC) working group. The SPFCIC was organized in 1940 and meets annually to address issues pertaining to forage production and management in the southeastern United States. Membership includes forage and animal scientists involved in forage education and research. Those LGU represented in the SPFCIC include Arkansas, Auburn, Clemson, Florida, Georgia, Kentucky, Louisiana State, Mississippi State, Missouri, North Carolina State, Oklahoma State, Texas A&M, and Virginia Tech.

The discussion and assessment were conducted from 27 January to 1 February. A one-question email was sent to the SPFCIC group regarding the training of forage agronomists; this was not a formal survey. The question asked was “*What is the average or typical number of M.S. and Ph.D. students graduated per year by your university in the subject matter of Forage Agronomy.”*

## RESULTS AND DISCUSSION

Thirteen LGU in the Southeastern United States were involved with the assessment; ten LGU responded for a 77% return rate. Responses from the SPFCIC assessment revealed an average of one MS student each year and one PhD student every 3 to 4 yr would graduate with an advanced degree in forage agronomy.

### Why So Few Forage Agronomists Trained?

Whole-animal research is needed to best study the ruminant-forage interface; however, it is expensive and overall budget limitations have reduced opportunities for whole-animal research. The whole-animal model allows researchers to examine relationships that exist among livestock, the forage component, and environment that cannot be examined individually. In addition to livestock costs, land availability and proper facilities to weigh and supplement cattle have made it difficult to conduct and publish research. Paddock or pasture is the replicated unit in most grazing studies; therefore, to conduct a statistically meaningful study requires greater land areas, as well as the infrastructure (fences, feed, and water troughs; DelCurto and Wyffels, 2021). To meet the minimum number of replications for significant power, some researchers must repeat studies across multiple years. The lack of replicated units and the environmental challenges, such as drought or pest invasion, results in many projects needing to be conducted over multiple years. This may limit the number of peer reviewed manuscripts grazing researchers can publish compared to other disciplines within the animal sciences that require small pens or individual animal as the experimental unit. This time commitment to conduct one study makes it difficult to train graduate students. Within the University of Arkansas and many other LGU, stipends for MS students are typically 2 yr in duration. This makes it arduous for a student to complete meaningful research unless there is an existing study available for the student to join immediately upon initiation of their program. Joining an existing research project limits the student’s ability to learn experimental design, as well as project planning and development. Factor in any environmental problems and the student may be delayed in the completion of their project. This uncertainty makes graduate work in forage agronomy unattractive to some potential students.

In addition to the concerns discussed above, there are fewer opportunities for funding in the field of forage agronomy. In states with predominant row crop cultivation, research geared toward agronomic crops is funded heavily by state and industry commodity groups. Additionally, in states with a greater livestock population and industry influence, industry and state commodity groups typically fund nutrition-based, end product (e.g., state Beef Council funding), and/or animal health-based research projects.

Faculty with different expertise should seek better opportunities for collaboration in the most current topics of research, teaching, and/or extension; however, forage agronomists may fall into a lower priority within Departments of Animal Science and Agronomy/Plant/Soil Sciences due to overall less funding for faculty positions. Faculty tends to be specialized to follow a research area/funding opportunity including more environmental stewardship and carbon sequestration in agronomy/crops/soil departments (Rouquette et al., 2009), and more health/immunology, wellbeing, and microbiome in the animal sciences. Departments typically will invest in areas that can achieve both external funding and peer-reviewed publications. Additionally, faculty in agronomy/crops/soil departments is more specialized in single species crops (e.g., soybean specialist, cotton specialist, and rice specialist), whereas forage agronomists must cover several forages species and be knowledgeable in livestock grazing, hay production, pest management, and environmental aspects.

Demographics of students have changed and students seem to be less familiar with the agronomic area. There are now more students from urban backgrounds and with interests other than traditional agriculture involving plants and/or animals. A phone survey conducted by Seed Your Future (https://www.seedyourfuture.org/), a group promoting horticulture and encouraging students to pursue careers working with plants, found 52% of U.S. citizens aged 18 to 34 were not familiar with the area of horticulture (Dole and Yoder, 2018). A majority of farms/ranches, specifically in the Southeastern United States, are small scale and less likely to implement intensive management resulting in diminished job demand specifically outside of LGU system. Greater funding of agronomic/crop/soil science departments by allied industry partners likely provides greater job opportunities for graduates studying grain crop production instead of forage production. Similarly, students in animal science departments also see greater job opportunities in the animal health and/or nutritional industries.

### Is Lack of Forage Agronomist Training an Education Issue Within the LGU?

Why are LGU in the western United States typically Departments of Animal and Range Sciences? Why are there no Departments of Animal and Forage Sciences located within LGU? Is it that rangeland systems are viewed as more “fragile” with one drought or overgrazing event away from a major disaster? Or is rangeland, being a limited resource thereby requiring more intensive management, result in the integration of the animal and plant sciences? Data from USDA (2020) show the three regions (Eastern, Central, and Western United States), had similar percentages of operations in which grazing lands make up at least 50% of the beef animal’s diet (96.6%, 95.0%, and 96.3% for Western, Central, and Eastern United States, respectively). Despite these similarities, greater percentages of operations rely on grazing cattle as the primary source of income in the Western and Central United States (15.8% and 23.5%, respectively) compared to 10.1% for the Eastern United States. Perhaps this greater percentage of operations grazing cattle in the Western and Central United States for income may require more intensive management, thereby requiring greater expertise in forage and range management compared to the Eastern U.S. Administrators at LGU should engage faculty and stakeholders to further investigate this dichotomy of programmatic structure within animal science department in the Eastern U.S.

### How Can We Improve Interest and Training in Forage Agronomy?

Introduction of forage agronomy and the livestock interaction earlier in the animal science curriculum is necessary. Furthermore, making plant identification a larger part of introductory courses will at least present the topic earlier and may result in improved interest in forage agronomy. We suggest students graduating with degrees in animal science should be able to identify the forage plants animals will consume just as they should be able to identify different livestock breeds. Other options would be to integrate pasture management into ruminant production courses including beef, dairy, and sheep production, and invite forage agronomists to lecture in these courses. Feeds and Feeding and Animal Nutrition courses within LGU typically focus on feedstuffs and the interaction with animal production. It would be beneficial to incorporate more forage/pasture components as part of these courses. In addition to normal ration formulation, exposing students to combined pasture and supplement formulation exercises would allow a greater appreciation of the contributions of feedstuffs and forages in grazing ruminant diets. Except for feedlot and dairy nutritionists, many commercial or consulting ruminant nutritionists will work with grazing cattle. Rather than have rangeland or forage-based courses as electives, animal science curriculum could be modified to require a minimum number of forage-related courses required for successful completion of an animal science degree. Finally, in departments with freshman or sophomore level courses in careers and professional development, inclusion of speakers with a forage agronomy background introduces forage agronomy earlier in their educational career.

Administrators and graduate program coordinators in Departments of Animal Science should proactively plan to make allowances for extending stipends due to the challenges of conducting grazing research. Considerations should be agreed upon prior to the acceptance of a graduate student focusing on forage agronomy that provides certainty funds, up to a point, would be available in cases of drought or other calamities out of the faculty/student’s control. This agreement reduces both student and faculty mentor anxiety, and potentially grows the number of forage agronomy graduate students. Cross-training of student across both animal science and agronomy/crop/soil departments is obviously something that should be considered and is the norm at certain universities. This allows for stipend, tuition, and research costs to be shared across departments. Expectations of each faculty member’s contributions, authorship on peer-reviewed manuscripts, etc., should be decided on among joint faculty in advance.

All programs need a better integration of all three of the LGU missions. Forage agronomy subject matter applies to both hay/silage production and to livestock grazing systems. However, both systems support livestock production. Unfortunately, forage agronomy students may not be exposed to the important animal production concepts during their graduate programs due to time constraints. This makes a steeper learning curve for students entering positions that emphasize grazing systems work. Integrating animal science and forage science extension faculty into graduate student programs can increase educational opportunities to help students understand application of their forage science training.

### Forage Agronomists Are Needed in Animal Science Departments

Most advertised faculty positions related to forages are typically outside the animal science department. Integration of a forage agronomist within animal sciences should result in a more robust beef cattle/forage program. Despite the need for this integration, forage agronomists may not be as successful as their animal science peers in publishing a similar number of studies on an annual basis. Therefore, forage agronomists must conduct small plot studies and do less field research to have a publication record needed for successful promotion. Grazing livestock studies are time and land/space consuming endeavors and may not allow generation of publications within a timeframe required for successful promotion. In addition to the challenges associated with publication, funding of forage sciences lags behind that of grain crops and animal health. Even though educational efforts of forage-based faculty may be on par with counterparts in traditional crop and animal sciences, lower generation of grants and program funding is often viewed with skepticism during the promotion review process. These obstacles may make it difficult for forage agronomists to find success in traditional animal science or agronomy/crops/soils departments.

Despite the previously noted challenges, it should be recognized that there is much opportunity for researchers in the forage/livestock area. Key word searches (forage, livestock, and grazing) in the *Journal of Animal Science, Agronomy Journal* and *Crop Science* conducted on January 6, 2022 show increasing numbers of published articles each decade since the 1960s (Table 1); it should be noted however that the majority of articles are within the *Journal of Animal Science*. The increased number of articles including these key words published from 2000 to 2019 in *Agronomy Journal and Crop Science* (Table 1) coincides with increased energy costs and the increased research interest regarding biofuels of the late 2000s (Rouquette et al., 2009), as well as research involving grazing as a method to reduce overall production cost due to high feed prices noted in that era (McCartney et al., 2008). In addition to biofuels, emerging issues such as mitigation of greenhouse gas emissions from ruminants with specialized forages, and supplements (Thompson and Rowntree, 2020), have allowed for greater research opportunities for researchers examining forage and livestock interactions. Moreover, newer technologies such as systems that automatically feed individual animals supplement in grazing situations have the potential to increase experimental units, thereby making completion and publication of grazing research more attainable (Husz et al., 2020), as well as systems that do not require traditional tools to capture body weight (Wells et al., 2021). These systems have the potential to individually weigh animals without traditional working facilities (chutes, tubs, and corrals) which can result in greater flexibility in research conducted on pastures/grazing lands. These new technologies coupled with the current issues show promise for greater opportunities for livestock forage agronomists. One thing to consider, these new technologies typically involve a yearly fee associated with data collection and management.

**Table 1.** Number of articles returned per decade from the *Journal of Animal Science*, *Agronomy Journal* and *Crop Science* using keywords “Forage, Livestock and Grazing”

Decade	Journal of Anim. Sci	Agronomy Journ.	Crop Sci.
1960–1969	37	70	21^a^
1970–1979	135	87	39
1980–1989	202	96	25
1990–1999	258	88	64
2000–2009	309	157	155
2010–2019	521	163	165
2020–2022^b^	71	73	38

*Crop Science* records began in 1961.

As of January 6, 2022.

We hypothesize that there are too few forage agronomists being trained, especially in light of newer opportunities in areas of research and other technologies. Animal science departments should strategically prioritize forage agronomy and highlight opportunities for students. Forage agronomists spend a majority of their time on livestock issues and tend to be a better “fit” in the animal sciences. Administration at LGU should engage faculty, industry stakeholders, and policy makers to explore avenues to increase the awareness and potential funding for training of forage agronomists.
